# Limitations in Evaluating Machine Learning Models for Imbalanced Binary Outcome Classification in Spine Surgery: A Systematic Review

**DOI:** 10.3390/brainsci13121723

**Published:** 2023-12-16

**Authors:** Marc Ghanem, Abdul Karim Ghaith, Victor Gabriel El-Hajj, Archis Bhandarkar, Andrea de Giorgio, Adrian Elmi-Terander, Mohamad Bydon

**Affiliations:** 1 Mayo Clinic Neuro-Informatics Laboratory, Mayo Clinic, Rochester, MN 55902, USA; marc.ghanem01@lau.edu (M.G.); ghaith.abdulkarim@mayo.edu (A.K.G.); victor.gabriel.elhajj@stud.ki.se (V.G.E.-H.); archis.bhandarkar@gmail.com (A.B.); bydon.mohamad@mayo.edu (M.B.); 2 Department of Neurological Surgery, Mayo Clinic, Rochester, MN 55902, USA; 3 School of Medicine, Lebanese American University, Byblos 4504, Lebanon; 4 Department of Clinical Neuroscience, Karolinska Institutet, 17177 Stockholm, Sweden; 5 Artificial Engineering, Via del Rione Sirignano, 80121 Naples, Italy; andrea@degiorgio.info; 6 Department of Surgical Sciences, Uppsala University, 75236 Uppsala, Sweden

**Keywords:** machine learning, artificial intelligence, deep learning, predictive modeling, spine surgery

## Abstract

Clinical prediction models for spine surgery applications are on the rise, with an increasing reliance on machine learning (ML) and deep learning (DL). Many of the predicted outcomes are uncommon; therefore, to ensure the models’ effectiveness in clinical practice it is crucial to properly evaluate them. This systematic review aims to identify and evaluate current research-based ML and DL models applied for spine surgery, specifically those predicting binary outcomes with a focus on their evaluation metrics. Overall, 60 papers were included, and the findings were reported according to the PRISMA guidelines. A total of 13 papers focused on lengths of stay (LOS), 12 on readmissions, 12 on non-home discharge, 6 on mortality, and 5 on reoperations. The target outcomes exhibited data imbalances ranging from 0.44% to 42.4%. A total of 59 papers reported the model’s area under the receiver operating characteristic (AUROC), 28 mentioned accuracies, 33 provided sensitivity, 29 discussed specificity, 28 addressed positive predictive value (PPV), 24 included the negative predictive value (NPV), 25 indicated the Brier score with 10 providing a null model Brier, and 8 detailed the F1 score. Additionally, data visualization varied among the included papers. This review discusses the use of appropriate evaluation schemes in ML and identifies several common errors and potential bias sources in the literature. Embracing these recommendations as the field advances may facilitate the integration of reliable and effective ML models in clinical settings.

## 1. Introduction

In recent years, the integration of machine learning (ML) into spine surgery has shown promise in enabling personalized risk predictions [1,2]. These advancements could improve patient outcomes, streamline surgical decision-making, reduce costs, and optimize medical management [3]. ML, a subset of artificial intelligence (AI), utilizes computer algorithms to efficiently solve intricate tasks. A notable advantage lies in its adaptability, enabling models to continually learn and be redesigned by incorporating new data and modifying their underlying knowledge.

Machine learning has witnessed significant advancements, notably in the realm of deep learning (DL)—an advanced subset that involves neural networks with multiple layers, enabling more intricate data processing and abstraction. This structure contributes to its capability to automatically learn and extract features from complex datasets [4]. The accumulation of advancements has garnered strong support from the industry, recognizing the substantial potential of ML and DL in enhancing medical research and clinical care [5]. However, despite the developments made in prediction models, their effective application in predicting uncommon outcomes remains limited in the literature. This brings attention to the class imbalance challenge in ML, where certain classes of interest occur far less frequently than others [6]. 

Imbalanced data essentially means that a dataset is skewed, leading to challenges with data generalizability, inadequate training of the ML model, and false positive readings. This issue is particularly relevant in medical ML models, where only a small proportion of individuals may experience a certain event, such as a specific condition or complication. In spine surgery, the outcomes of interest, such as readmission, extended length of stay, or specific complications, are considered infrequent events. In such cases, the integration of ML for personalized risk predictions becomes trickier, as the rarity of these specific events adds complexity to predictive modeling. If ML models lack design considerations for tackling class imbalance, they may become skewed towards one end of the spectrum, making their predictions unreliable. This underscores the significance of addressing the class imbalance challenge within ML. Hence, this review highlights the importance of refining our understanding and application of evaluation methods to navigate the complexities of uncommon outcome predictions more effectively.

## 2. Inadequate Evaluation Metrics

A classifier can only be as effective as the metric used to assess it. Selecting the wrong metric for model evaluation can lead to suboptimal model training or even mislead the authors into selecting a poor model instead of a better-performing one. Below are metrics that should not be solely relied on for imbalanced classification.

### 2.1. Accuracy

Accuracy measures how well a model predicts the correct class. It is calculated as the ratio of correct predictions to the total number of predictions. However, when evaluating a binary classification model on an imbalanced dataset, accuracy can be misleading. This is because it only considers the total number of correct predictions without weighing the dataset’s imbalance.

In scenarios with imbalanced datasets, a model consistently predicting the majority class can exhibit high accuracy but may struggle to accurately identify the minority class. When accuracy closely aligns with the class imbalance rate, it suggests the model might be predicting the majority class for all instances. In such cases, the accuracy is driven by the class imbalance, hindering the model’s ability to distinguish between positive and negative classes. Therefore, it is crucial to employ multiple metrics for a comprehensive evaluation of the model’s performance.

### 2.2. The Area under the ROC Curve (AUROC)

AUROC is calculated as the area under the curve of the true positive rate (TPR) versus the false positive rate (FPR). A no-skill classifier will have a score of 0.5, whereas a perfect classifier will have a score of 1.0.

While AUROC is useful for comparing the performance of different models, it can be misleading with class imbalance as the TPR and FPR are affected by the class distribution.

For instance, in a model predicting a specific disease on an imbalanced dataset, the TPR may be low as the model struggles to predict sick cases, while the FPR may be high because the model accurately predicts healthy cases. In such instances, the AUROC may yield falsely high-performance results.

### 2.3. Adequate Evaluation Metrics

In assessing a binary classification model on an imbalanced dataset, key metrics include the confusion matrix (CM), F1 score, Matthews correlation coefficient (MCC), and area under the precision-recall curve (AUPRC).

### 2.4. Confusion Matrix

The CM matrix delineates true positive, true negative, false positive, and false negative in model predictions [7]. This matrix is particularly useful for imbalanced classes, offering insights into the model’s performance on each class separately. It also facilitates the calculations of various metrics such as precision, recall, and F1 score. 

As mentioned earlier, relying solely on accuracy is advised against in imbalanced cases, with the confusion matrix providing a strong rationale for that. Researchers can use it to visualize the model’s performance, pinpoint common errors, and make the necessary adjustments to enhance overall performance. Table 1 displays the metrics provided by the CM.

### 2.5. F1 Score

Improving the model’s performance often involves aiming for a balance between precision and recall. However, it is essential to acknowledge that there is a trade-off between these two metrics, where enhancement of one metric score can lead to a reduction in the other. The correct balance is highly reliant on the model’s objective and is referred to as the F1 score. The F1 score is particularly useful when faced with imbalanced classes as it emphasizes the harmonic mean between precision and recall [8].

### 2.6. Matthews Correlation Coefficient (MCC)

The Matthews correlation coefficient (MCC) stands out as a robust metric, especially when dealing with imbalanced class data. MCC is a balanced metric that takes into account all four components of the CM. It is defined as (TP × TN − FP × FN)/sqrt((TP + FP) × (TP + FN) × (TN + FP) × (TN + FN)). The MCC tends to approach +1 in cases of perfect classification and −1 in instances of entirely incorrect classification (inverted classes). When facing class-imbalanced data, the MCC is considered a strong metric due to its effectiveness in capturing various aspects of classification performance. Notably, it remains close to 0 for completely random classifications.

### 2.7. Informedness (Youden’s J Statistic)

Informedness, also known as Youden’s J statistic, quantifies the difference between the true positive rate (Recall) and the false positive rate (FPR). It is computed as Recall + Specificity − 1, with values ranging from −1 to +1. A higher informedness value signifies a superior classifier [9].

### 2.8. Markedness

Markedness gauges the difference between the PPV and NPV. The calculation involves adding PPV and NPV, then subtracting 1, resulting in a range from −1 to +1. A higher markedness value suggests a better overall performance in predictive values [9].

### 2.9. The Area under the Precision-Recall Curve (AUPRC)

AUPRC is a valuable metric when working with imbalanced datasets as it considers precision and recall in its calculation [10]. This is important when dealing with imbalanced datasets where the focus is on identifying positive cases and minimizing false positives. The AUPRC is derived by plotting precision and recall values at various thresholds and then computing the area under the resulting curve.

The resulting curve is formed by different points, and classifiers performing better under different thresholds will be ranked higher. On the plot, a no-skill classifier manifests as a horizontal line with precision proportional to the number of positive examples in the dataset. Conversely, a point in the top right corner signifies a perfect classifier.

### 2.10. Brier Score (BS)

The Brier Score (BS) serves as a metric for assessing the accuracy of a probabilistic classifier and is used to evaluate the performance of binary classification models [11]. It is determined by calculating the mean squared difference between the predicted probabilities for the positive class and the true binary outcomes. The BS ranges from 0 to 1, with a score of 0 indicating a perfect classifier, while 1 suggests predicted probabilities completely discordant with actual outcomes. 

It is important to note that while the BS possesses desirable properties, it does have limitations. For instance, it may favor tests with high specificity in situations where the clinical context requires high sensitivity, especially when the prevalence is low [12].

To address these limitations, a model’s BS evaluation should consider the outcome prevalence in the patient sample, prompting the computation of the null BS. The null BS acts as a benchmark for evaluating a model’s performance by always predicting the most prevalent outcome in the dataset. The model’s BS is then compared to that of the null model, and ΔBrier is calculated by subtracting the null BS from that of the model under evaluation. The ΔBrier is a scalar value and indicates the extent to which the model outperforms the null model. The formula follows ΔBrier = BS of the model − BS of the null model.

### 2.11. Additional Evaluation Metrics and Graphical Tools

#### 2.11.1. Calibration Curves

A calibration plot is a graphical tool used to evaluate a probabilistic model. The curve illustrates the alignment between the model’s predicted probabilities and the observed frequencies of the positive class in the test set. A perfect model would exhibit an intercept value of 0 and a slope value of 1. These plots are particularly valuable for evaluating models trained on imbalanced data, offering insights into the model’s ability to predict the positive class.

Addressing imbalanced data involves using techniques such as undersampling and oversampling to achieve classification balance and alleviate classifier bias. However, determining the optimal sample size for training remains a significant challenge. An alternative strategy is to leverage learning curves, which provide insights into reducing error probability as the training set size increases. One example is a theoretical learning curve for the multi-class Bayes classifier, considering general multivariate parametric models of class-conditional probability density [13]. This curve offers an estimate of the reduction in the excess probability of error without relying on specific model parameters. Learning curves contribute to an essential understanding of the model’s behavior and its performance improvements with increased data. Table 1 outlines the metrics derived from the confusion matrix.

#### 2.11.2. Decision Curve

**Figure 1:**
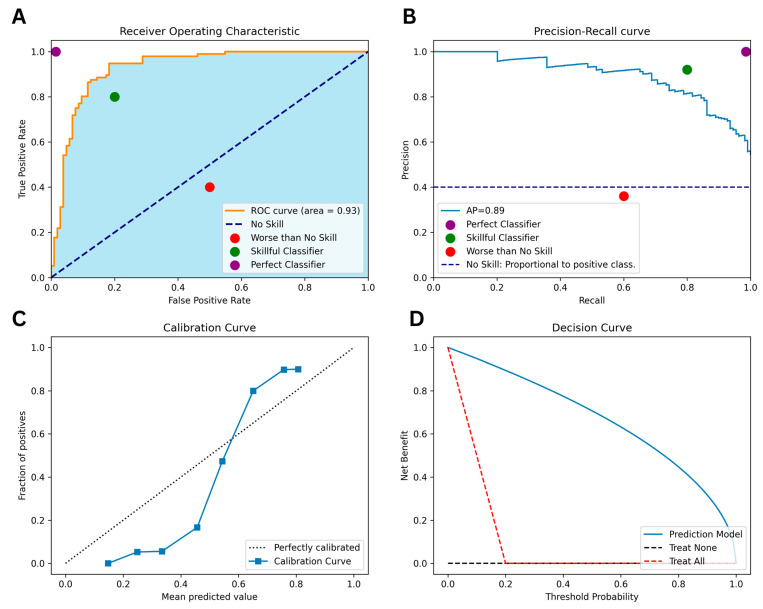
Illustrations of Various Performance Metrics for the Same Classifier: (**A**) Area Under the Receiver Operating Characteristic Curve, (**B**) Area Under the Precision-Recall Curve, (**C**) Calibration Curve, (**D**) Decision Curve.

A decision curve is a graphical tool used to evaluate a classifier’s performance by examining the trade-off between sensitivity and 1-specificity across varying thresholds for classifying an instance as positive. The optimal threshold is the one that maximizes the net benefit. By convention, the model’s benefit strategy at each threshold is compared to the treat-all and treat-none strategies. The decision curve analysis stands out from other statistical methods by its ability to evaluate the clinical value of a predictor. Figure 1A–D depicts the AUROC, AUPRC, calibration, and decision curve figures.

With that in mind, this systematic review of the literature aims to provide a comprehensive summary of the state of AI within the field of spine surgery. The focus will be on reporting metrics, data visualization, and common errors, including inappropriate handling of imbalanced datasets and incomplete reporting of model performance metrics.

## 3. Materials and Methods

### 3.1. Data Sources and Search Strategies

**Figure 2:**
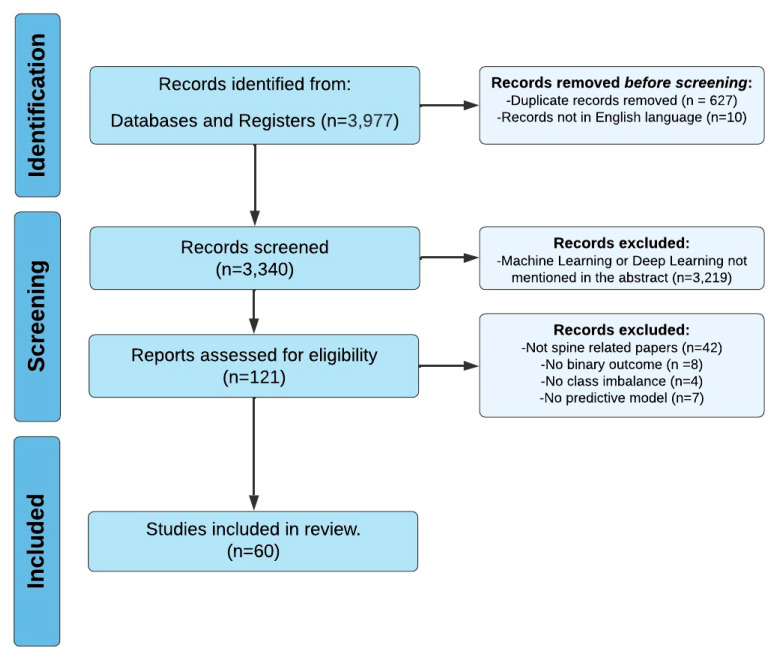
PRISMA Flowchart Illustrating Systematic Review Search Strategy.

A comprehensive search of several databases was performed on 28 February 2023. Results were limited to the English language but had no date limitations. The databases included Ovid MEDLINE(R), Ovid Embase, Ovid Cochrane Central Register of Controlled Trials, Ovid Cochrane Database of Systematic Reviews, Web of Science Core Collection via Clarivate Analytics, and Scopus via Elsevier. The search strategies were designed and conducted by a medical librarian in collaboration with the study investigators (Table S1). Controlled vocabulary supplemented with keywords was used. The actual strategies listing all search terms used and how they are combined are available in the Supplemental Material. Ultimately, 3340 papers and 121 full-text articles were assessed, resulting in the inclusion of 60 studies (Figure 2) [14,15,16,17,18,19,20,21,22,23,24,25,26,27,28,29,30,31,32,33,34,35,36,37,38,39,40,41,42,43,44,45,46,47,48,49,50,51,52,53,54,55,56,57,58,59,60,61,62,63,64,65,66,67,68,69,70,71,72]. This review was conducted in accordance with the PRISMA guidelines (Table S2).

### 3.2. Eligibility Criteria and Data Extraction

Inclusion criteria encompass studies focusing on ML-based prediction models pertaining to binary surgical outcomes following spine surgery. Both intraoperative and postoperative outcomes were eligible. Exclusion criteria comprised studies predicting nonbinary outcomes (e.g., 3+ categorical or numeric outcomes), those predicting non-spine surgical outcomes, studies with balanced outcomes, and those lacking predictive models.

The extracted data from all studies included the first author, paper title, year of publication, spinal pathology and surgery type, sample size, outcome variable (the primary result being measured), imbalance percentage, accuracy, AUROC (area under the receiver operating characteristic curve), sensitivity, specificity, PPV (positive predictive value), NPV (negative predictive value), Brier score (BS), other metrics, dataset, performance, journal, and error type (Table 2).

### 3.3. Data Synthesis and Risk of Bias Assessment

Our aim was to investigate the methodologies employed by the included studies, emphasizing the process rather than the outcomes or findings themselves. Accordingly, we refrained from engaging in narrative synthesis, data pooling, risk of bias assessment, or evidence certainty determination. Instead, our review specifically addressed methodologies related to models handling class imbalance. 

### 3.4. Statistical Analysis

Given the considerable heterogeneity between studies, we did not perform a meta-analysis and opted for a qualitative and comprehensive analysis instead. Study characteristics are presented using frequencies and percentages for categorical variables. In cases where studies reported multiple results within a single outcome (e.g., different AUCs per type of complication), the top scores were taken. Metrics were computed for studies that provided a confusion matrix.

## 4. Results

### 4.1. Characteristics of the Included Studies

**Figure 3:**
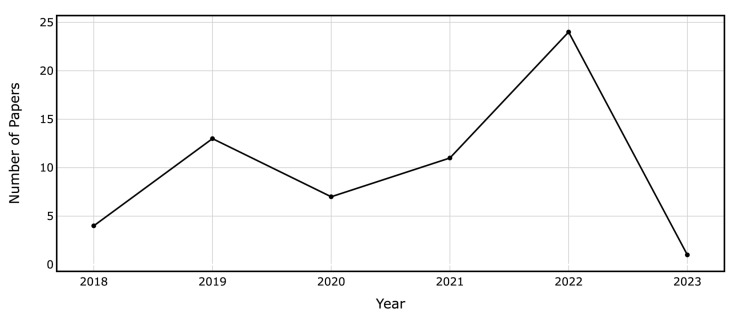
Annual Count of ML and DL Papers on Binary Outcome Prediction in Spine Surgery Included in the Review.

The selected papers cover a variety of outcomes, some focusing on a single target while others address multiple targets. Table 2 outlines the metrics derived from the confusion matrix. Among the 60 papers, 12 focused on readmissions, 13 predicted lengths of stay (LOS), 12 addressed non-home discharge, 6 estimated mortality, and 5 anticipated reoperations. The models also forecasted a variety of medical and surgical outcomes, as detailed in Table 3. The target outcomes exhibited data imbalances ranging from 0.44% to 42.4%. Figure 3 illustrates the growing number of papers in the field over time.

In the analysis of the 60 included papers, 59 reported the model’s AUROC, 28 mentioned accuracies, 33 provided sensitivity, 29 discussed specificity, 28 addressed PPV, 24 considered NPV, 25 indicated BS (with 10 providing null model Brier), and 8 detailed the F1 score. Additionally, a variety of representations and visualizations were presented in these papers: 52 included an AUROC figure, 27 featured a calibration curve, 13 displayed a confusion matrix, 12 showcased decision curves, 3 incorporated PR curves, and only 1 offered a precision-recall curve. Moreover, to train their models, 23 studies utilized NSQIP data, and 19 used single-center data, while the rest used multicenter data or other national datasets. In the following sections, we explore prevalent errors observed in the reviewed articles, highlighting key areas for improvement in the evaluation and reporting of machine learning models in spine surgery applications.

### 4.2. Error Type I: Incomplete Reporting of Performance Metrics

Han et al. presented models predicting various medical and surgical complications, demonstrating strong performance metrics such as AUROCs, BS, sensitivity, and acceptable specificity [15]. Similarly, Arora et al. developed a well-performing model that predicts patient discharge to rehabilitation, achieving high AUROC, sensitivity, and specificity with an adjusted threshold of 0.16 [32]. Both studies also demonstrated well-calibrated models through calibration plots.

Shah et al. developed models predicting readmission or major complications, achieving satisfactory AUROC, AUPRC, and BS while outperforming the baseline AUPRC, indicating its effectiveness in predicting true positives well [17]. Valliani et al. predicted non-home discharge with remarkable AUROCs, PPV, and NPV. The study also presented a well-calibrated model through a calibration plot, although the plot did not display true probability and predicted risks greater than 0.8 [18]. Despite these models’ solid performance on the metrics reported, studies in this category failed to report other metrics crucial for model evaluation. While some omitted the PPV and NPV, others failed to mention baseline AUPRC, sensitivity, specificity, and the null model BS. Without the inclusion of all the necessary evaluation metrics, the assessment lacks validity, even when reported metrics show high performance.

### 4.3. Error Type IIA: Metric Optimization at the Expense of Others

Li et al. developed artificial neural networks (ANN) and random forest (RF) models for predicting day-of-surgery patient discharge. The ANN model exhibited high sensitivity but low specificity, while the RF model showed the opposite [26]. Kim et al. and Arvind et al. presented models predicting mortality, wound complications, venous thromboembolism, and cardiac complications [30,31,34]. The Linear regression (LR) models exhibited high specificities at the expense of extremely low sensitivities. In contrast, ANN displayed high sensitivities and specificities but low PPVs. Goyal et al. developed models predicting non-home discharge and 30-day unplanned readmission [24]. The models predicting non-home discharge achieved high AUROCs, accuracies, sensitivity, specificity, and NPV but low PPV, leading to many false positives. This training method is advised only when the target is critically important and should not be missed, even if it means encountering many false positives.

Stopa et al. and Karhade et al. trained models to predict non-routine discharge, presenting high AUROC, BS, specificity, and NPV but low sensitivity and PPV [21,25]. Although both models demonstrated well-calibrated performance via calibration plots, they struggled to detect positive cases correctly, facing low sensitivity scores and PPVs. Moreover, both papers presented a decision curve demonstrating that their models are better than the treat-all or the treat-non approach.

### 4.4. Error Type IIB: High Accuracy and AUROC but Poor Sensitivity

Cabrera et al. developed models that predict extended LOS, readmission, reoperation, infection, and transfusion. Although these models achieved high accuracies, their sensitivities were generally low, except for the model predicting transfusion [14]. Gowd et al. predicted multiple surgical outcomes with high AUROCs and NPV but low PPV and extremely low sensitivity scores [19]. Kalagara et al. trained models to predict unplanned readmission, reporting high accuracies but low sensitivities, while specificity, PPV, and NPV were not provided [22]. Hopkins et al. developed a readmission prediction model with high accuracy, AUROC, specificity, PPV, and NPV but low sensitivity, indicating an inability to identify a significant proportion of true positive instances [23].

### 4.5. Other Errors

In addition to the previously mentioned errors, some papers provided poor calibration plots and omitted essential metrics. Kuris et al., Veeramani et al., and Zhang et al. presented models predicting readmission, unplanned re-intubation, and short LOS, respectively, with acceptable AUROCs, accuracies, and BSs [16,27,29]. However, all three studies provided calibration plots indicating poor calibration, as the calibration curves were not in proximity to the near-perfect prediction diagonal. Moreover, the null model BS was not reported. Ogink et al. developed models predicting non-home discharge displaying adequate AUROCs and BSs [33]. Nevertheless, the calibration plots in both studies revealed that the models were not well-calibrated for larger observed proportions and predicted probabilities, as the calibration curves drifted away from the near-perfect prediction diagonal. Furthermore, these five papers failed to report sensitivities, specificities, PPVs, and NPVs.

## 5. Discussion

ML’s ability to predict future events by training on vast healthcare data has attracted substantial interest [73]. Nevertheless, predicting rare events poses significant challenges attributed to the skewed data distribution. To address this issue, techniques for imbalanced class learning have been designed. This paper focuses on showcasing the application of ML in predicting uncommon patterns or events within the realm of spinal surgeries. These surgeries encompass various risks and require a thorough assessment of potential outcomes, such as readmission, reoperation, ELOS, and discharges to non-home settings [74,75].

We reviewed 60 papers addressing post-spinal surgery outcome predictions, examining specific elements of spinal surgeries such as pathologies, surgical procedures, and spinal levels. However, a limited number of these studies adequately evaluated their models using suitable metrics for imbalanced data binary classification tasks. This observation highlights the need for more rigorous model evaluation methods to ensure their clinical reliability and effectiveness in rare-event predictions. In a study by Haixiang et al., it was revealed that 38% of the 517 papers addressing imbalanced classification across various domains used accuracy as an evaluation metric despite the authors’ awareness of dealing with an imbalanced problem [76]. In some instances, the accuracy of a proposed method might be lower than the class imbalance ratio, implying that a dummy classifier solely predicting the majority class would yield better performance.

The importance of appropriate evaluation metrics for imbalanced classification problems in machine learning cannot be overstated. Our analysis revealed that many papers relied on inadequate evaluation metrics. Moreover, our review identified instances where models optimized one metric at the expense of others. These practices can lead to misinterpretation of model performance and hinder clinical applicability. Therefore, it is crucial to conduct a comprehensive evaluation of classifier performance, addressing all relevant metrics rather than focusing on only one or two. Additionally, striking a balance between the various performance metrics is essential to ensure that models can be effectively employed in clinical decision-making. By emphasizing the need for a holistic approach to classifier evaluation, our study encourages the development of more robust and reliable ML models for predicting rare outcomes in spinal surgery and other healthcare applications.

Training a binary classification model on an imbalanced dataset, where one class significantly outnumbers the other, poses challenges as the model may be biased towards the more prevalent class. Most strategies addressing this issue can be applied in the preprocessing stage prior to model training. These strategies include undersampling the majority class, oversampling the minority class, modifying weights, and optimizing thresholds.

Undersampling involves reducing instances of the majority class in the training sample to equalize the classes. Various undersampling techniques, such as random undersampling, NearMiss, cluster-based undersampling, and Tomek links, can balance a dataset. Random undersampling selects a subset of majority class examples randomly, while NearMiss retains examples from the majority class closest to the minority class [77]. Cluster-based undersampling sorts majority class examples into clusters and selects a representative subset from each cluster. Tomek links remove examples from the majority class closely related to minority class examples, increasing the space between classes and facilitating classification [78].

Another method for balancing classes is oversampling, which entails adding more minority class examples to the training dataset. For binary classification, strategies such as random oversampling, the synthetic minority over-sampling technique (SMOTE), and adaptive synthetic sampling (ADASYN) can be employed. Random oversampling adds random minority class samples to the training set until classes are equal, potentially leading to overfitting if the oversampled data does not represent the original minority class distribution. SMOTE, a more advanced technique, creates synthetic samples using the k-nearest neighbors algorithm to ensure new samples resemble original minority class samples [79]. ADASYN is similar to SMOTE but generates synthetic samples more representative of the feature space region where the minority class is under-represented. While oversampling techniques appear more promising than undersampling ones, especially with small datasets, it is important to note that oversampling involves the addition of synthetic data that might not correspond to the real data. Given this constraint, advanced generative deep-learning algorithms were developed [80,81]. One such advancement is generative adversarial network synthesis for oversampling (GANSO), which has demonstrated superior performance compared to the synthetic minority oversampling technique (SMOTE) [82].

In addition to the sampling methods discussed, threshold optimization can enhance classification model performance by adjusting the decision threshold for identifying positive category cases [83]. This involves calculating the model’s performance at various thresholds and selecting the one with the best performance. It is essential to conduct this optimization on a separate validation set to avoid overfitting. Once the optimal threshold is determined, it can be applied to a model’s predictions on new data.

It is good practice to systematically test various suitable algorithms for the task at hand. Decision tree algorithms, such as random forest (RF), classification and regression tree (CART), and C4, perform well with imbalanced datasets. Additionally, classifiers’ performance can be enhanced by assigning weights based on the inverse of class frequencies or using advanced techniques like cost-sensitive learning. In place of traditional classification models, anomaly detection models can also be used. Ensemble methods, such as bagging and boosting, are also effective in handling imbalanced data. Finally, it is crucial to evaluate using appropriate metrics for imbalanced classification tasks, such as MCC, CM, precision, recall, F1 score, and AUPRC. By employing a diverse set of metrics and considering the unique characteristics of each dataset, researchers can avoid being misled by metrics like accuracy and AUROC. 

## 6. Conclusions

This systematic review summarizes the current literature on ML and DL in spine surgery outcome prediction. Evaluating these models is crucial for their successful integration into clinical practice, especially given the imbalanced nature of spine surgery predicted outcomes. The 60 papers reviewed focused on binary outcomes such as ELOS, readmissions, non-home discharge, mortality, and reoperations. The review highlights the prevalent use of the AUROC metric in 59 papers. Other metrics like sensitivity, specificity, PPV, NPV, Brier score, and F1 score were inconsistently reported.

Based on the findings of this review, our recommendations for future research in ML applications for spine surgery are threefold. First, we advocate for the comprehensive use and reporting of all appropriate evaluation metrics to ensure a holistic assessment of model performance. Second, developing strategies to optimize classifier performance on imbalanced data is crucial. Third, we stress the necessity of increasing awareness among researchers, reviewers, and editors about the pitfalls associated with inadequate model evaluation. To improve peer review quality, we suggest including at least one ML specialist in the review process of medical AI papers, as a high level of model design scrutiny is not a realistic demand from clinician reviewers.

The significance of proper evaluation schemes in applied ML cannot be overstated. Embracing these recommendations as the field advances will undoubtedly facilitate the integration of reliable and effective ML models in clinical settings. Ultimately, integrating such models in the clinical setting will contribute to improved patient outcomes, surgical decision-making, and medical management in spine surgery.

## Figures and Tables

**Table 1:** Metrics Provided by the Confusion Matrix.

Metrics Provided by the Confusion Matrix.	
True Positive (TP)	The number of predictions where the classifier correctly predicts the positive class as positive.
True Negative (TN)	The number of predictions where the classifier correctly predicts the negative class as negative.
False Positive (FP)	The number of predictions where the classifier incorrectly predicts the negative class as positive.
False Negative (FN)	The number of predictions where the classifier incorrectly predicts the positive class as negative.
Recall/Sensitivity	The proportion of true positive predictions to all actual positive cases TP/(TP + FN).
Specificity	The proportion of all negative samples that are correctly predicted as negative by the classifier TN/(TN + FP).
Precision/Positive predictive value (PPV)	The proportion of true positive predictions to all positive predictions TP/(TP + FP).
Negative predictive value (NPV)	The proportion of true negative predictions to all negative predictions made by the model TN/(TN + FN).

**Table 2:** Performance Metrics, Datasets, and Outcome Variables in Reviewed ML Studies on Imbalanced Binary Classification in Spine Surgery.

Author	Year	Primary Pathology and Surgery Type	Sample Size	Outcome Variable	Imbalance	Accuracy	AUROC	Sensitivity	Specificity	PPV	NPV	Brier	Other Metric	Dataset	Performance Related Figures	Journal	Error Type
Cabrera	2022	Posterior Cervical Decompression with Instrumented Fusion	29,949	>4 days LOS	18.21% (5454)	0.781	0.781	0.4978	0.842	-	-	-	-	NSQIP 2008–2018	AUROC Calibration plot	Journal of Clinical Neuroscience	I and II
				Readmission	4.4% (1318)	0.9512	0.791	0.4615	0.9718			-					
				Reoperation	2.51% (752)	0.9559	0.781	0.4333	0.9683			-					
				Infection	4.4% (1318)	0.9311	0.724	0.1695	0.9676			-					
				Transfusion	2.6% (779)	0.7577	0.902	0.8864	0.7532			-					
Han	2019	Spine Surgery	345,510 * 760,724 **	Pulmonary complications	4.7% (16,138) * 5.3% (40,046) **	-	0.75	0.82	0.52	-	-	0.044	-	MKS */CMS **	AUROC Calibration plot	The Spine Journal	I and II
				Congestive heart failure	1.0% (3538) * 3.6% (26,989) **	-	0.75	0.84	0.51	-	-	0.026					
				Pneumonia	1.9% (6629) * 2.9% (21,861) **	-	0.74	0.81	0.51	-	-	0.024					
				Urinary tract infections	3.3% (11,410) * 6.2% (46,786) **	-	0.71	0.78	0.52	-	-	0.075					
				Neurologic complications	2.1% (7317) * 4.0% (29,462) **	-	0.69	0.76	0.51	-	-	0.032					
				Cardiac dysrhythmia	4.3% (14,689) * 10.6% (80,822) **	-	0.72	0.78	0.53	-	-	0.53					
				Overall adverse events	18.0% (60,958) * 27.6% (209,646) **	-	0.7	0.71	0.57	-	-	0.166					
				Overall medical complications	-	-	0.7	-	-	-	-	-					
				Overall surgical complications	-	-	0.69	-	-	-	-	-					
Kuris	2021	Anterior, Posterior, and Posterior Interbody Lumbar Spinal Fusion	63,533 ALIF: 12,915 PLIF: 27,212 PSF:23,406	Readmission	ALIF: 4.92% (635) PLIF: 4.41% (1200) PSF: 4.49% (1051)	0.94–0.95	0.64–0.65	-	-	-	-	0.048–0.052	-	NSQIP 2009–2018	Visualization of BS Calibration plot	World Neurosurgery	I
Shah	2021	Lumbar Spinal Fusion	38,788	Readmission or Major Complication	11.5% (4470)	-	0.686	-	-	-	-	0.094	AUPRC: 0.283	All California hospitals 2015–2017	AUROC PR-curve	World Neurosurgery	I
Valliani	2022	Thoracolumbar Spine Surgery	SCDW: 5224	Non-home discharge	SCDW: 23.28% (1216)	-	0.81	-	-	0.64	0.83	-	-	Algorithm development: SCDW *** 2008–2019	AUROC Calibration plot	World Neurosurgery	I
			NIS:492,312		NIS: 20.64% (101,613)	-	0.77	-	-	0.6	0.82	-	-	Out-of-sample validation: National Inpatient Sample 2009–2017			
Gowd	2022	Anterior Cervical Discectomy and Fusion	42,194	Any adverse event	3.14% (1327)	-	0.73	0.029	0.9994	0.615	0.966	-	-	NSQIP 2011–2017	AUROC Confusion matrix	World Neurosurgery	II
				Extended length of stay	16.36% (6905)	-	0.73	0.1821	0.9793	0.65	0.85	-	-				
				Transfusion	0.44% (184)	-	0.9	0.0294	0.9998	0.4	0.996	-	-				
				Surgical site infection	058% (243)	-	0.63	0	1	0	0.993	-	-				
				Return to OR	1.58% (667)	-	0.64	0	1	0	0.982	-	-				
				Pneumonia	0.76% (3210)	-	0.8	0.0102	0.9989	0.067	0.992	-	-				
Ogink	2019	Spondylolisthesis Surgery	9338	Non-home discharge	18.6% (1737)	-	0.753	-	-	-	-	0.132 Null: 0.152	-	NSQIP 2009–2016	AUROC Calibration plot	European Spine Journal	I
Karhade	2018	Lumbar Degenerative Disc Disorders Elective Surgery	26,364	Non-routine discharge	9.28% (2447)	-	0.823	-	-	0.33	0.54	0.0713 Null: 0.086	-	NSQIP 2011–2016	AUROC Calibration plot Decision curve	Neurosurgical Focus	I
Kalagara	2019	Lumbar Laminectomy	26,869	Unplanned readmission	5.59% (1502)	0.950/0.796	0.801/0.690	0.496/0.405	-	-	-	-	-	NSQIP 2011–2014	-	J Neurosurg Spine	I and II
Hopkins	2020	Posterior Lumbar Fusion	23,264	Readmission	5.15% (1198)	0.962	0.812	0.355	0.995	0.785	0.97	-	-	NSQIP 2011–2016	AUROC	J Neurosurg Spine	II
Goyal	2019	Spinal Fusion	59,145	Discharge to non-home facility	12.6% (7452)	0.77–0.79	0.85–0.87	0.77–0.80	0.77–0.79	0.32–0.35	0.96	-	-	NSQIP 2012–2013	-	J Neurosurg Spine	II
				30-day unplanned readmission	4.5% (2662)	0.59–0.71	0.63–0.66	0.46–0.63	0.59–0.72	0.07	0.97	-	-				
Stopa	2019	Elective Spine Surgery	144	Non-routine discharge	6.9% (10)	-	0.89	0.6	0.95	0.5	0.97	0.049	-	**** 2013–2015	AUROC Calibration plot Decision curve Confusion matrix	J Neurosurg Spine	II
Li	2022	Single-Level Laminectomy Surgery	35,644	Discharged on day of surgery	37.1% (13,230)	0.69/0.70	0.77/0.77	0.83/0.58	0.55/0.80	0.77/0.69	0.64/0.70	-	-	NSQIP 2017–2018	-	Global Spine Journal	II
Veeramani	2022	Anterior Cervical Discectomy and Fusion	54,502	Unplanned re-intubation	0.51% (278)	72–99.6	0.52–0.77	-	-	-	-	0.04–0.18	-	NSQIP 2010–2018	AUROC Calibration plot	Global Spine Journal	I
DiSilvestro	2020	Metastatic Intraspinal Neoplasm Excision	2094	Mortality	5.16% (108)	-	0.898	-	-	-	-	-	-	NSQIP 2006–2018	AUROC	World Neurosurgery	I
Zhang	2021	Posterior Spine Fusion Surgery	1281	Short LOS	20.5% (262)	0.68–0.83	0.566–0.821	-	-	-	-	0.13–0.29	-	NSQIP 2006–2018	AUROC Calibration plot	Journal of Clinical Medicine	I
Kim	2018	Posterior Lumbar Spine Fusion	22,629	Cardiac complications	0.44% (100)	-	0.71	0	0.9997	0	0.9985	-	-	NSQIP 2010–2014	AUROC Confusion matrix	Spine (Phila Pa 1976)	I and II
				VTE complications	1.06% (242)	-	0.588	-	-	-	-	-	-				
				Wound complications	1.86% (420)	-	0.613	0	0.9999	0	0.9785	-	-				
				Mortality	0.15% (34 )	-	0.703	-	-	-	-	-	-				
Arvind	2018	Anterior Cervical Discectomy	20,879	Mortality	0.1% (21)	-	0.979	0.1667	0.9943	0.0278	0.9992	-	-	Multicenter data set & NSQIP 2010–2014	AUROC Confusion matrix	Spine Deformity	I and II
				Wound complications	0.5% (105)	-	0.518	0.5429	0.4458	0.0055	0.9943	-	-				
				VTE complications	0.3% (63)	-	0.656	-	-	-	-	-	-				
				Cardiac complications	0.2% (42)	-	0.772	-	-	-	-	-	-				
Arora	2022	Elective Spine Surgery	3678	Discharged to rehabilitation	22% (809)	-	0.79	0.8	0.64	-	-	-	-	Single academic institution	AUROC	Spine Epidemiology	I
Ogink	2019	Lumbar spinal stenosis	28,600	Non-home discharge	18.2% (5205)	-	0.751	-	-	-	-	0.131 Null: 0.15	-	NSQIP 2009–2016	AUROC Calibration plot	European Spine Journal	I
Kim	2018	Spinal Deformity Procedures	4073	Mortality	0.5% (29)	-	0.844	0	1	0	0.9937	-	-	NSQIP 2010–2014	AUROC Confusion matrix	Spine Deformity	I & II
				Wound complications	2.4% (139)	-	0.606	0.6579	0.5871	0.0343	0.9872	-	-				
				VTE complications	1.8% (105)	-	0.547	-	-	-	-	-	-				
				Cardiac complications	0.7% (39)	-	0.768	-	-	-	-	-	-				
Zhang	2022	Degenerative spinal disease surgery	663	Postop Delerium	27.45% (182)	0.77	0.87	0.861	0.773	-	-	-	F1: 0.673 Youden: 0.34	Single academic institution	Calibration plots Decision curve	CNS Neuroscience & Therapeutics	I
Yang	2022	Thoracolumbar burst fracture	161	Perioperative blood loss	38.5% (62)	0.783	0.864	0.867	0.814	0.741	0.826	-	F1: 0.793	Single academic institution	AUROC	Frontiers in Public Health	None
Xiong	2022	Posterior Lumbar Interbody Fusion	584	Surgical site infection	5.65% (33)	0.9107	0.8726	0.3333	0.974	0.625	0.9184	-	F3: 0.5747	Single academic institution	AUROC Confusion matrix	Computational & Mathematical Methods in Medicine	II
Wang	2020	Microvascular decompression	912	Postop Delerium	24.2% (221)	0.923	0.962	0.788	-	0.881	-	-	F1: 0.832	Single academic institution	AUROC	Journal of Clinical Anesthesia	I
Wang	2021	Posterior Lumbar Fusion	13,500	Venous thromboembolism	0.95% (1283)	-	0.709	-	-	-	-	-	-	NSQIP 2010–2017	-	Global Spine Journal	I
Wang	2021	Posterior laminectomy and fusion with cervical myelopathy	184	C5 palsy	14.13% (26)	0.918	0.923	0.6667	0.9677	0.8	0.9375	-	-	Single academic institution	AUROC Confusion matrix	Journal of Orthopaedic Surgery and Research	None
Wang	2021	Minimally Invasive Transforaminal Lumbar Interbody Fusion	705	Surgical site infections	4.68% (33)	0.9	0.78	-	-	-	-	-	-	Single academic institution	AUROC	Frontiers in Medicine	I
Zhang	2021	Posterior Spine Fusion Surgery	1281	Short length of stay	20.5% (262)	0.831	0.814	-	-	-	-	0.13	-	NSQIP 2006–2018	AUROC Calibration plots	Journal of neurosurgery	I
Valliani	2022	Cervical Spine Surgery	SAI: 4342 NIS: 311,582	Extended length of stay	25% (1086/77,896)	-	0.87/0.84	0.70/0.57	0.89/0.92	0.75/0.75	0.86/0.83	-	-	Single academic institution National Inpatient Sample	AUROC	Neurosurgery	None
Stopa	2019	Elective Spine Surgery	144	Non-routine discharge	6.9% (10)	-	0.89	-	-	0.5	0.97	-	-	**** 2013–2015	AUROC Calibration plot	Neurosurgery	I
Siccoli	2019	Lumbar spinal stenosis	635	Reoperation Overall	9.5% (60)	0.69	0.66	0.32	0.69	0.1	0.9	0.09	F1: 0.15	Single academic institution	AUROC	Neurosurgical Focus	II
			635	Reoperation at Index	4.3% (27)	0.63	0.61	0.5	0.64	0.07	0.96	0.05	F1: 0.12				
			451	Prolonged Operation	15% (68)	0.78	0.54	0.85	0.23	0.91	0.14	0.13	F1: 0.88				
			633	Extended Hospital Stay	15% (95)	0.77	0.58	0.27	0.87	0.28	0.86	0.13	F1: 0.27				
Shah	2022	Posterior cervical spinal fusion	6822	Major complication or 30-day readmission	18.8% (1279)	0.7214	0.679	0.5117	0.7699	0.3394	0.8722	0.4081	AUPRC: 0.377	California hospitals 2015- 2017	AUROC PR-curve Confusion matrix	European Spine Journal	II
Saravi	2022	Lumbar Decompression Surgery	236	Extended length of stay	25% (59)	0.814	0.814	-	-	-	-	-	-	Single academic institution	AUROC	Journal of Clinical Medicine	I
Russo	2021	Anterior Cervical Discectomy and Fusion	1516	Extended length of stay	42.4% (643)	0.66/0.69	0.68/0.68	0.52/0.49	0.72/0.78	0.44/0.48	0.78/0.78	-	-	Single academic institution	AUROC Confusion matrix Decision curve	Journal of the American Academy of Orthopaedic Surgeons	II
Rodrigues	2022	Anterior Cervical Discectomy and Fusion	176,816	2-yr reoperation	5.6% (9956))	-	0.671	-	-	-	-	-	-	^ 2007 to 2016	AUROC Calibration plot	Spine	I
				90-day complication	7.5% (13,254)		0.823										
				90-day readmission	6.3% (11,192)		0.713										
Ren	2022	Lumbar Discectomy	1159	Recurrent lumbar disc herniation	11.22% (130)	0.8641	-	0.8269	-	0.8958	-	-	F1: 0.86	Single academic institution	AUROC	Global Spine Journal	I
Porche	2022	Lumbar surgery	231	Urinary retention	25.9% (60)	-	0.737	0.954	0.254	0.6	0.79	-	-	Single academic institution	AUROC Confusion matrix Calibration plot	Journal of Neurosurgery Spine	I
Pedersen	2022	Lumbar Disc Herniation	1988	EuroQol	36.5% (726)	0.79	0.84	0.7	0.84	0.83	0.71	-	MCC ^^: 0.54 F1: 0.83	Danish national registry for spine surgery	-	Global Spine Journal	None
				Oswestry Disability Index	36.3% (721)	0.69	0.74	0.67	0.7	0.71	0.65	-	MCC ^^: 0.37 F1: 0.71				
				Visual Analog Scale Leg	32.3% (643)	0.64	0.65	0.43	0.8	0.66	0.6	-	MCC ^^: 0.25 F1: 0.57				
				Visual Analog Scale Back	32.3% (643)	0.72	0.78	0.64	0.77	0.79	0.61	-	MCC ^^: 0.41 F1: 0.78				
				Ability to return to work (1 year)	14.2% (282)	0.86	0.81	0.61	0.92	0.91	0.63	-	MCC ^^: 0.53 F1: 0.91				
Nunes	2022	Thoracolumbar fractures surgery	215,999	30-day readmission	8.8% (19,148)	0.575	0.743	0.776	0.556	0.145	0.962	-	F1: 0.245	HCUP and SID in 187 hospitals in Florida 2014 to 2018	-	International Journal of Health Planning & Management	II
Merali	2019	Degenerative cervical myelopathy	605	6 Month: SF-6D	-	0.718	0.71	0.75	0.5	0.9	0.25	-	-	Multicenter AOSpine CSM North America	AUROC Confusion matrix	PLoS ONE	II
				12 Month: SF-6D		0.77	0.7	0.78	0.63	0.98	0.12						
				24 Month: SF-6D		0.708	0.73	0.74	0.47	0.92	0.17						
				6 Month: mJOA		0.667	0.73	0,7	0.59	0.82	0.43						
				12 Month: mJOA		0.713	0.73	0.7	0.59	0.82	0.43						
				24 Month: mJOA		0.649	0.67	0.63	0.8	0.96	0.23						
Martini	2021	Spine Surgery	11,150	Non-home discharge	15.8% (1764)	-	0.91	-	-	-	-	-	-	Single academic institution	AUROC	Spine	I
Khan	2020	Degenerative Cervical Myelopathy	702	Worsening functional status	12.1% (85)	0.714	0.788	0.779	0.704	-	-	-	-	Multicenter	AUROC Calibration plot	Neurosurgery	I
Karhade	2019	Spinal metastasis	1790	30-day mortality	8.49% (152)	-	0.769	-	-	-	-	0.0706 Null: 0.079	-	NSQIP 2009 through 2016	AUROC Calibration plot Decision curve	Neurosurgery	I
Karhade	2019	Lumbar disc herniation	5413	Sustained postoperative opioid prescription	7.7% (416)	-	0.79	-	-	-	-	0.065 Null: 0.071	-	Multicenter	AUROC Calibration plot Decision curve	The Spine Journal	I
Karhade	2019	Anterior cervical discectomy and fusion	2737	Sustained postoperative opioid prescription	9.9% (270)	-	0.8	-	-	-	-	0.075 Null: 0.089	-	Multicenter	AUROC Calibration plot Decision curve	The Spine Journal	I
Karhade	2022	Spinal metastasis	4303	6-week mortality	14.17% (610)	-	0.84	-	-	-	-	0.1 Null: 0.12	-	Multicenter	AUROC Calibration plot Decision curve	The Spine Journal	I
Karhade	2019	Lumbar spine surgery	8435	Sustained postoperative opioid prescription	2.5% (82)	-	0.7	-	-	-	-	0.039 Null: 0.041	-	Multicenter	AUROC Calibration plot Decision curve	The Spine Journal	I
Karhade	2021	Anterior lumbar spine surgery	1035	Intraoperative vascular injury	7.2% (75)	-	0.92	0.86	0.93	0.52	0.99	0.04 Null: 0.077	F1: 0.44 AUPRC: 0.74	Multicenter	AUROC Calibration plot Decision curve	The Spine Journal	II
							0.75	-	-	-	-	0.072 Null: 0.077	-				I
Karhadea	2021	Anterior cervical discectomy and fusion	2917	Length of stay greater than one day	35.2% (1027)	-	0.68	-	-	-	-	0.21	-	-	AUROC Calibration plot	Seminars in Spine Surgery	I
Karabacak	2023	Spinal Tumor Resections	3073	Prolonged length of stay	25% (769)	0.804	0.745	0.618	-	0.478	-	-	F1: 0.538 MCC: 0.422 AUPRC: 0.602	NSQIP 2015 through 2020	AUROC PR-curve	Cancers	II
				Non-home discharge	23.4% (718)	0.75	0.701	0.442	-	0.375	-	-	F1: 0.405 MCC: 0.250 AUPRC: 0.408				II
				Major complications	12.33% (379)	0.856	0.73	0.383	-	0.221	-	-	F1: 0.279 MCC: 0.216 AUPRC: 0.309				II
Jin	2022	Intradural Spinal Tumors	4488	Readmission	11.7% (524)	-	0.693/ 0.525/ 0.643	-	-	-	-	0.093/ 0.093/ 0.099	-	IBM MarketScan Claims Database 2007–2016	AUROC Calibration plots	Neurospine	I
				Non-home discharge	18.9% (956)	-	0.786	-	-	-	-	0.155					
Jain	2020	Long Segment Posterior Lumbar Spine Fusion	37,852	Discharge-to-facility	35.4% (13,400)	-	0.77	-	-	-	-	-	-	State Inpatient Database 2005–2010	AUROC	The Spine Journal	I
				90-day readmission	19.0% (7192)	-	0.65	-	-	-	-	-	-				
				90-day major medical complications	13.0% (4921)	-	0.7	-	-	-	-	-	-				
Hopkins	2020	Posterior spinal fusions	4046	Surgical Site Infection	1.5% (61)	-	0.775	0.4955	0.9988	0.9256	0.985	-	-	Single academic institution	AUROC	Clinical Neurology & Neurosurgery	II
Fatima	2020	Lumbar Degenerative Spondylolisthesis	80,610	Overall adverse events	4.9% (3965)	-	0.7	-	-	-	-	-	-	NSQIP 2005–2016	AUROC Calibration plot Decision curve	World Neurosurgery	I & II
				Medical adverse events	10.1% (8165)	-	0.7	-	-	-	-	0.02	-				
				Surgical adverse events	1.9% (1518)	-	0.69	-	-	-	-	0.07	-				
				Pneumonia	0.6% (450)	-	0.71	0.95	0.91	0.26	-	0.04	-				
				Bleeding transfusion	5.3% (4268)	-	0.7	0.98	0.95	0.24	-	0.05	-				
				Urinary tract infection	1.3% (1074)	-	0.7	-	-	-	-	0.01	-				
				Superficial wound infection	0.9% (750)	-	0.62	0.97	0.95	0.23	-	-	-				
				Sepsis	0.6% (473)	-	0.63	-	-	-	-	-	-				
Etzel	2022	Lumbar Arthrodesis	ALIF:12,915 PLIF/TLIF: 27,212 PSF: 23,406	Prolonged length of stay	-	0.799/ 0.813/ 0.804	0.752/ 0.723/ 0.753	-	-	-	-	0.15/ 0.15 0.14	-	NSQIP 2009–2018	AUROC Calibration plots	Journal of the American Academy of Orthopaedic Surgeons	I
Elsamadicy	2022	Metastatic Spinal Column Tumors	4346	Readmission	22.8% (991)	-	0.59	-	-	-	-	-	-	Nationwide Readmission Database 2016–2018	AUROC	Global Spine Journal	I
Dong	2022	Minimally Invasive Kyphoplasty in Osteoporotic Vertebral Compression Fractures	346	Risk of Recollapse	11.56% (40)	0.8844	0.81	0.875	0.8856	0.5	0.9819	-	-	Single academic institution	AUROC Confusion matrix	Frontiers in Public Health	II
Dong	2022	Lumbar Interbody Fusion	157	Short Term Unfavorable Clinical Outcomes	16.56% (26)	0.9367	0.88	0.7667	0.9766	0.8846	0.947	-	-	Single academic institution	AUROC Confusion matrix	BMC Musculoskeletal Disorders	None
				Long Term Unfavorable Clinical Outcomes	5.7% (9)	0.9459	0.78	0.9291	0.9776	0.9874	0.8792	-	-				
Yen	2022	Lumbar disc herniation	1316	Sustained postoperative opioid prescription	3.1% (41)	-	0.76	-	-	-	-	0.028	AUPRC: 0.33	Single academic institution	AUROC AUPRC Calibration plot Decision curve	The Spine Journal	I

* Truven MarketScan (MKS) and MarketScan Medicaid Databases; ** Centers for Medicare and Medicaid Services (CMS) Medicare database. *** Single-center data warehouse; **** Transitional Care Program at Brigham and Women’s Hospital. ^ IBM MarketScan Commercial Claims and Encounters Database and Medicare Supplement; ^^ Matthews’s correlation coefficient. HCUP: Healthcare Cost and Utilization Project; PR: Precision-Recall; SID: State Inpatient Database; AUROC: Area under the ROC curve; AUPRC: Area under the PR curve; BS: Brier Score.

**Table 3:** Outcome variables predicted by ML models in reviewed studies.

Topic	Complication	Number
Infection	Surgical site infection	5
	Wound complications	3
	Infection	1
	Sepsis	1
General Adverse Events	Surgical adverse events	2
	Any adverse event	4
	Major complications	1
	Medical adverse events	5
	Mortality	6
	Readmission	12
	Reoperation	5
Quality of Life/Pain	Visual Analog Scale Back	1
	Visual Analog Scale Leg	1
	6 Month: mJOA	1
	6 Month: SF-6D	1
	12 Month: mJOA	1
	12 Month: SF-6D	1
	Sustained postoperative opioid prescription	4
	24 Month: mJOA	1
	24 Month: SF-6D	1
	EuroQol	1
	Ability to return to work (1 year)	1
	Worsening functional status	1
	Oswestry Disability Index	1
Surgical	Risk of Recollapse	1
	Prolonged Operation	1
	Recurrent lumbar disc herniation	1
	Intraoperative vascular injury	1
Cardiac	Cardiac complications	3
	Cardiac dysrhythmia	1
	Congestive heart failure	1
Pulmonary	Pulmonary complications	1
	Unplanned re-intubation	1
	Pneumonia	3
Length of Stay	Extended length of stay	10
	Short length of stay	3
Neurology	C5 palsy	1
	Neurologic complications	1
	Postop delerium	2
Other	VTE complications	4
	Transfusion	3
	Perioperative blood loss	1
	Urinary retention	1

## Data Availability

No new data were created or analyzed in this study. Data sharing is not applicable to this article.

## Supplementary Materials

The following supporting information can be downloaded at https://www.mdpi.com/article/10.3390/brainsci13121723/s1, Table S1: Search strategy; Table S2: PRISMA 2020 checklist.
