# Supplementary material for: Limitations in Evaluating Machine Learning Models for Imbalanced Binary Outcome Classification in Spine Surgery: A Systematic Review
Source: Brain Sci. 2023 Dec 16;13(12):1723. doi: 10.3390/brainsci13121723 (PMC10741524; doi:10.3390/brainsci13121723)
Supplement: Supplementary file 1 [file brainsci-13-01723-s001.zip › S1, Search strtegy.pdf]

Search Strategies

OVID

Database(s): **Embase** 1988 to 2023 Week 08**, Ovid MEDLINE(R) and Epub Ahead of Print, In-Process, In-Data-Review & Other Non-Indexed Citations** 1996 to February 27, 2023**, EBM Reviews - Cochrane Central Register of Controlled Trials** January 2023**, EBM Reviews - Cochrane Database of Systematic Reviews** 2005 to February 22, 2023
Search Strategy:

#	Searches
1	exp artificial intelligence/
2	("artificial intelligence*" or "deep learning" or "machine learn*" or "neural network*" or "network learning" or "1-nearest neighbor" or "a-i" or AdaBoost or ai or "association rule learning" or "automated pattern recognition" or "Bayesian learning" or "bayesian network*" or "computational intelligence*" or "computer reason*" or "computer vision*" or "connectionist model*" or "decision tree*" or "deep unified network*" or "expert system*" or "fuzzy logic*" or "fuzzy system*" or "genetic algorithm*" or heuristic* or "hidden markov model*" or "inductive logic program*" or "kernel method*" or "K-nearest neighbor" or "knowledge base*" or "knowledge engineering" or (knowledge and (acquisition* or aquir* or represent*) and (computer* or machine)) or knowledgebase* or (learn* adj2 algorithm*) or "learning classifier system*" or "learning machine*" or "logistic model tree*" or "machine intelligence*" or "metric learning" or "multilayer perceptron*" or "naive bayes" or "naive bayesian classifier*" or "natural language processing*" or perceptron* or radiomic* or "random forest*" or "random survival forest*" or "RBF network*" or "reinforcement learning" or "relevance vector machine*" or "representation learning" or "self-organizing map*" or "similarity learning" or "sparse dictionary learning" or "supervised learning" or "support vector machine*" or "unsupervised learning").ti,ab,hw,kf.
3	(algorithm* or automat* or computer*).ti.
4	or/1-3
5	exp Spinal Fusion/
6	exp Spine/su
7	exp Spinal Diseases/su
8	Lumbosacral Region/su
9	((spine or spinal or lumbar or lumbosacral or cervico* or cervical or thoracic or thoracolumbar or occipitocervical or "occipito-cervical" or occipitalcervical or "occipital-cervical" or atlantoaxial or "atlanto-axial" or occipitoatlantoaxial or "occipoto-atlantoaxial" or "craniovertebral junction" or subluxation or vertebr* or intervertebral or

	disc or discs or disk or disks or sacral or sacrum or "trans-sacr*") adj10 (fusion* or fused or fusing or fixation or decompression or surg* or operat* or arthrodesis or reconstruct*)).ti,ab,hw,kf.
10	(discectom* or diskectom* or laminectomy or spondylosyndesis).ti,ab,hw,kf.
11	exp Decompression, Surgical/
12	spinal cord decompression/
13	exp Diskectomy/
14	or/5-13
15	4 and 14
16	limit 15 to english language [Limit not valid in CDSR; records were retained]
17	limit 16 to yr="2010 -Current"
18	remove duplicates from 17

SCOPUS

1	TITLE-ABS-KEY ("artificial intelligence*" OR "deep learning" OR "machine learn*" OR "neural network*" OR "network learning" OR "1-nearest neighbor" OR "a-i" OR adaboost OR ai OR "association rule learning" OR "automated pattern recognition" OR "bayesian learning" OR "bayesian network*" OR "computational intelligence*" OR "computer reason*" OR "computer vision*" OR "connectionist model*" OR "decision tree*" OR "deep unified network*" OR "expert system*" OR "fuzzy logic*" OR "fuzzy system*" OR "genetic algorithm*" OR heuristic* OR "hidden markov model*" OR "inductive logic program*" OR "kernel method*" OR "k-nearest neighbor" OR "knowledge base*" OR "knowledge engineering" OR (knowledge AND (acquisition* OR aquir* OR represent*) AND (computer* OR machine)) OR knowledgebase* OR (learn* W/2 algorithm*) OR "learning classifier system*" OR "learning machine*" OR "logistic model tree*" OR "machine intelligence*" OR "metric learning" OR "multilayer perceptron*" OR "naive bayes" OR "naive bayesian classifier*" OR "natural language processing*" OR perceptron* OR radiomic* OR "random forest*" OR "random survival forest*" OR "rbf network*" OR "reinforcement learning" OR "relevance vector machine*" OR "representation learning" OR "self-organizing map*" OR "similarity learning" OR "sparse dictionary learning" OR "supervised learning" OR "support vector machine*" OR "unsupervised learning")

2	TITLE-ABS-KEY ((spine OR spinal OR lumbar OR lumbosacral OR cervico* OR cervical OR thoracic OR thoracolumbar OR occipitocervical OR "occipito-cervical" OR occipitalcervical OR "occipital-cervical" OR atlantoaxial OR "atlanto-axial" OR occipitoatlantoaxial OR "occipoto-atlantoaxial" OR "craniovertebral junction" OR subluxation OR vertebr* OR intervertebral OR disc OR discs OR disk OR disks OR sacral OR sacrum OR "trans-sacr*") W/10 (fusion* OR fused OR fusing OR fixation OR decompression OR surg* OR operat* OR arthrodesis OR reconstruct*)) OR TITLE-ABS-KEY (discectom* OR diskectom* OR laminectomy OR spondylosyndesis)
3	1 AND 2
4	INDEX(embase) OR INDEX(medline) OR PMID(0* OR 1* OR 2* OR 3* OR 4* OR 5* OR 6* OR 7* OR 8* OR 9*)
5	3 NOT 4
6	DOCTYPE(ed) OR DOCTYPE(bk) OR DOCTYPE(er) OR DOCTYPE(no) OR DOCTYPE(sh) OR DOCTYPE(ch)
7	5 NOT 6
8	7 AND (PUBYEAR > 2009) AND LIMIT-TO (LANGUAGE , "English"))

Web of Science

1	TS=("artificial intelligence*" OR "deep learning" OR "machine learn*" OR "neural network*" OR "network learning" OR "1-nearest neighbor" OR "a-i" OR adaboost OR ai OR "association rule learning" OR "automated pattern recognition" OR "bayesian learning" OR "bayesian network*" OR "computational intelligence*" OR "computer reason*" OR "computer vision*" OR "connectionist model*" OR "decision tree*" OR "deep unified network*" OR "expert system*" OR "fuzzy logic*" OR "fuzzy system*" OR "genetic algorithm*" OR heuristic* OR "hidden markov model*" OR "inductive logic program*" OR "kernel method*" OR "k-nearest neighbor" OR "knowledge base*" OR "knowledge engineering" OR (knowledge AND (acquisition* OR aquir*

	OR represent*) AND (computer* OR machine)) OR knowledgebase* OR (learn* NEAR/2 algorithm*) OR "learning classifier system*" OR "learning machine*" OR "logistic model tree*" OR "machine intelligence*" OR "metric learning" OR "multilayer perceptron*" OR "naive bayes" OR "naive bayesian classifier*" OR "natural language processing*" OR perceptron* OR radiomic* OR "random forest*" OR "random survival forest*" OR "rbf network*" OR "reinforcement learning" OR "relevance vector machine*" OR "representation learning" OR "self-organizing map*" OR "similarity learning" OR "sparse dictionary learning" OR "supervised learning" OR "support vector machine*" OR "unsupervised learning")
2	TI=((spine OR spinal OR lumbar OR lumbosacral OR cervico* OR cervical OR thoracic OR thoracolumbar OR occipitocervical OR "occipito-cervical" OR occipitalcervical OR "occipital-cervical" OR atlantoaxial OR "atlanto-axial" OR occipitoatlantoaxial OR "occipoto-atlantoaxial" OR "craniovertebral junction" OR subluxation OR vertebr* OR intervertebral OR disc OR discs OR disk OR disks OR sacral OR sacrum OR "trans-sacr*") NEAR/10 (fusion* OR fused OR fusing OR fixation OR decompression OR surg* OR operat* OR arthrodesis OR reconstruct*)) OR TS=(discectom* OR diskectom* OR laminectomy OR spondylosyndesi
3	1 AND 2
4	PMID=(0* or 1* or 2* or 3* or 4* or 5* or 6* or 7* or 8* or 9*)
5	3 NOT 4
6	5 and PY=2010-2023
7	Refined By:Languages: English
